# Performance of Three Asthma Predictive Tools in a Cohort of Infants Hospitalized With Severe Bronchiolitis

**DOI:** 10.3389/falgy.2021.758719

**Published:** 2021-10-22

**Authors:** Ronaldo C. Fabiano Filho, Ruth J. Geller, Ludmilla Candido Santos, Janice A. Espinola, Lacey B. Robinson, Kohei Hasegawa, Carlos A. Camargo

**Affiliations:** ^1^Emergency Medicine Network, Department of Emergency Medicine, Massachusetts General Hospital, Boston, MA, United States; ^2^Division of Rheumatology, Allergy and Immunology, Massachusetts General Hospital, Boston, MA, United States; ^3^Department of Emergency Medicine, Harvard Medical School, Boston, MA, United States

**Keywords:** asthma, bronchiolitis, predictive tools, infancy, children, wheezing

## Abstract

Childhood asthma develops in 30–40% of children with severe bronchiolitis but accurate prediction remains challenging. In a severe bronchiolitis cohort, we applied the Asthma Predictive Index (API), the modified Asthma Predictive Index (mAPI), and the Pediatric Asthma Risk Score (PARS) to predict asthma at age 5 years. We applied the API, mAPI, and PARS to the 17-center cohort of infants hospitalized with severe bronchiolitis during 2011–2014 (35th Multicenter Airway Research Collaboration, MARC-35). We used data from the first 3 years of life including parent interviews, chart review, and specific IgE testing to predict asthma at age 5 years, defined as parent report of clinician-diagnosed asthma. Among 875/921 (95%) children with outcome data, parent-reported asthma was 294/875 (34%). In MARC-35, a positive index/score for stringent and loose API, mAPI, and PARS were 24, 68, 6, and 55%, respectively. The prediction tools' AUCs (95%CI) ranged from 0.57 (95%CI 0.54–0.59) to 0.68 (95%CI 0.65–0.71). The positive likelihood ratios were lower in MARC-35 compared to the published results from the original cohorts. In this high-risk population of infants hospitalized with severe bronchiolitis, API, mAPI, and PARS had sub-optimal performance (AUC <0.8). Highly accurate (AUC >0.8) asthma prediction tools are desired in infants hospitalized with severe bronchiolitis.

## Introduction

Asthma is a chronic inflammatory airway condition affecting ~8% of US children (1) and is projected to cost over $300 billion from 2018 to 2038 (2). Nonetheless, asthma pathobiology is incompletely understood (3–5). Childhood asthma development often precedes recurrent or severe asthma-like symptoms, especially wheezing (6–9). Bronchiolitis is the leading cause of US infants' hospitalization (7, 9, 10). Among these infants, ~30-40% will develop childhood asthma (7, 10–13). The accurate identification of those at higher risk is important to optimize preventive strategies and treatment.

Several asthma prediction scores are used in children (14). Common elements include frequency of wheezing episodes, atopic symptoms/diagnosis, parents' asthma history, and objective measurement of atopy, alongside clinical and laboratory data (14, 15). Despite these similarities, the scores have shown variable performance in predicting asthma development (14, 16). For this analysis, we included (1) the first and most used model, the Asthma Predictive Index (API) (17), developed in the Tucson Children's Respiratory Study (TCRS) (18); (2) the modified Asthma Predictive Index (mAPI) (19), developed in The Prevention of Asthma in Kids (PEAK) trial and validated in the Childhood Origins of ASThma (COAST) study (20); and (3) the Pediatric Asthma Risk Score (PARS) (21), developed in the Cincinnati Childhood Allergy and Air Pollution Study (CCAAPS) (22). The API and mAPI tools were validated in clinical settings (23, 24) while PARS has not yet been externally replicated (16).

We investigated the prediction performance of API, mAPI, and PARS in our prospective cohort of children hospitalized with severe bronchiolitis, using data from infancy to age 3 years to predict asthma development at age 5 years.

## Materials and Methods

### Study Design

We analyzed data from the 35th Multicenter Airway Research Collaboration (MARC-35), a multi-center, prospective cohort of infants hospitalized for bronchiolitis. MARC-35 is coordinated by the Emergency Medicine Network (EMNet), a collaboration of 247 hospitals. During the 2011–2014 winter seasons, investigators at 17 hospitals enrolled infants (age <1 year) hospitalized with an attending physician diagnosis of bronchiolitis (see Appendix in the Supplementary Material) (25). Infants born at <32 gestational weeks and those with known heart-lung disease were excluded. All parents/guardians provided written informed consent. The study was approved by the institutional review board at all participating hospitals.

### Data Collection

During the hospitalization, investigators conducted structured interviews with parents to assess the infants' familial, environmental, and medical history, and details of the bronchiolitis episode. Additional data on clinical course and treatments were collected via medical record abstraction. Blood samples were collected within 24 h of hospitalization for measurement of serum specific IgE (sIgE) (26).

Follow-up data collection is conducted by the EMNet Coordinating Center at the Massachusetts General Hospital. Participants are followed via biannual parent telephone interviews, which include detailed history of breathing problems, breathing medications, and development of asthma and allergic conditions. The present analysis used data from birth to age 5 years. We conducted an examination during early childhood, which included a complete blood count with differential and collection of blood samples for serum sIgE measurement. The exam was conducted during ages 3–5.9 years. For the present analysis, we only used exam data collected up to age 4.9 years, excluding 38/611 (6%) children whose exam occurred at age ≥5 years. Additionally, we conducted a structured medical record abstraction for infancy (age <1 year).

### Parent-Reported Data

Definitions of predictor variables, outcomes, and prediction rules used in API, mAPI, and PARS in their original cohorts and MARC-35 are provided in the Supplementary Table 1. Parent-reported data collected at enrollment included infant sex and race/ethnicity and parental history of asthma. At enrollment and biannual interviews, eczema was assessed with the question, “Does [child] have a history of eczema, also known as atopic dermatitis? By “eczema,” we mean an itchy, scale rash that comes and goes.” We defined eczema as parent report of eczema by age 3 years (27, 28). At the age 3-year interview, allergic rhinitis was assessed with the questions, “Has your child ever had a problem with sneezing, or a runny, or blocked nose when he/she DID NOT have a cold or the flu?” and “Has your child ever had hayfever?” We defined allergic rhinitis as an affirmative response to either question (27, 29).

At each interview, parents were asked if the child had experienced any breathing problems since the previous interview, characterized by severe coughing, breathing faster or harder than normal, wheezing, or shortness of breath. For each breathing problem, parents reported the duration, whether it was related to an acute respiratory infection, whether the child visited a healthcare provider, and medication use (inhaled corticosteroids, systemic corticosteroids, inhaled bronchodilator, and montelukast) in the days surrounding the episode. We used these data to define early frequent wheeze as parent report of ≥3 breathing problems during ages 1–1.9 years or 2–2.9 years (stringent API), and parent report of ≥4 breathing problems which lasted at least 1 day, at least one of which was associated with a healthcare visit, during age 2–2.9 years (mAPI). We defined wheezing apart from colds as parent report of at least one breathing problem which was not related to an acute respiratory infection during ages 1–2.9 years (API, mAPI) or during ages 0–2.9 years (PARS). All MARC-35 participants were considered to have met the loose API and PARS criterion of “early wheeze” due to their bronchiolitis hospitalization during infancy.

### Blood Eosinophilia

We used blood eosinophil data from two sources, the infancy medical record and the early childhood exam, to approximate the data collection time frames for eosinophilia in API (mean age 11 months) and mAPI (age range 24–48 months). Eosinophilia was defined as ≥4% eosinophils observed in the age <1 year medical record (API) or at the early childhood exam (mAPI). Eosinophilia during age <1 year included data from the enrollment hospitalization and pre-hospitalization emergency department or clinic visits. The proportion of participants with eosinophilia data measured at the same age as reported in the API (before 1 year of age) was 773/863 (89.6%) for the stringent API and 783/875 (89.5%) for the loose API. For the mAPI, if eosinophil data from the early childhood exam were unavailable, we used data from the age 1–3.5 year medical record (if available; *n* = 26) or age <1 year (*n* = 262).

### Allergic Sensitization

We examined allergic sensitization via sIgE at enrollment and the early childhood exam using two different assays (ImmunoCAP sIgE and ImmunoCAP ISAC) at the Phadia Immunology Reference Laboratory (Portage, MI). The sIgE food allergen assays at enrollment (26) and the early childhood exam included milk, egg white, peanut, cashew nut, and walnut. Additionally, in the early childhood sera only, we conducted sIgE assays for aeroallergens, including grasses, trees, weeds, molds, cat, dog, cockroach, mouse, and dust mites (*D. farinae* and *D. pteronyssinus*). Positive sIgE results were defined as ≥0.35 kU/L. The ImmunoCAP ISAC microarray immunoassays at enrollment and the early childhood exam included 112 components representing 19 food allergens, 10 aeroallergens (grasses, weeds, trees, cat, dog, horse, mouse, molds, dust mites, and cockroach), and three other allergens (latex, venom, and anisakis) with positive results defined as ≥0.30 ISU-E (ISAC Standardized Units). We defined food and aeroallergen sensitization (mAPI) as sensitization to any food or aeroallergen, respectively, via sIgE or the ISAC chip at the early childhood exam. If IgE data from the early childhood exam were unavailable, we used data from the age 1–4.9 year medical record (if available; *n* = 10) or age <1 year (*n* = 338). We defined polysensitization (PARS) as sensitization to ≥2 food allergens or aeroallergens across infancy, the early childhood exam, and the age 1–4.9 year medical record. Participants who were missing IgE data from early childhood and were not polysensitized (*n* = 325; based on the data from infancy and the age 1–4.9 years medical record) were considered missing for this PARS criterion, as children generally become sensitized to more allergens by age 3 years (30).

### Asthma Ascertainment

During biannual interviews starting at age 30 months, parents were asked if the child had ever been told by a doctor or other health professional that he/she has asthma, and if so, at what age. We defined asthma at age 5 years as parent report of clinician-diagnosed asthma from any interview between ages 30 months and 5 years. Our asthma definition has been validated against medical record review (31). In the validation study, this asthma definition had 80% sensitivity, 92% specificity, 86% positive predictive value, 88% negative predictive value, and 0.86 AUC compared to medical record view confirming physician-diagnosed asthma (31).

### Statistical Analysis

We calculated the prevalence of predictor variables and asthma in MARC-35. We compared the characteristics of children who developed asthma at age 5 years to those who did not, using chi-square tests and Kruskal-Wallis tests, as appropriate. We applied the stringent API, loose API, mAPI, and PARS to MARC-35 data from infancy to age 3 years to predict asthma at age 5 years. Scores were calculated as described in their original publications (Supplementary Table 1). For the stringent API, loose API, and mAPI, infants were included if they had the minimum amount of data necessary to ascertain a positive index (i.e., data on early frequent wheeze and at least one major criterion or two minor criteria for stringent API). For PARS, we used all available data, and dichotomized scores as <7 vs. ≥7. We calculated the test characteristics (sensitivity, specificity, positive predictive value [PPV], negative predictive value [NPV], positive and negative likelihood ratios [LR+ and LR–, respectively], and area under the receiver operating characteristic curve [AUC] with 95% confidence intervals [95%CI]) of the prediction tools in MARC-35, and compared the observed test characteristics to the published performance of the tools in their original cohorts (17–22). We considered AUC >0.8 as the threshold of strong performance.

To assess the relative importance of variables used to predict asthma within each score, we used two approaches: (1) we calculated the AUC of each predictor variable to predict asthma at age 5 years, using all available data, and (2) we obtained adjusted odds ratios (ORs) and 95%CIs from multivariable logistic regression models for asthma at age 5 years, adjusted for all predictor variables in the respective prediction tool.

We conducted sensitivity analyses to assess the robustness of the main results. (1) We excluded infants who were born preterm (32–37 gestational weeks). (2) We applied the tools using complete data only; participants who were missing data on any criterion for a tool were excluded from the analysis of that tool. (3) We used eosinophilia data from the early childhood exam for the API eosinophilia criterion, as eosinophil values measured during infancy may have been affected by the bronchiolitis episode. (4) We used a narrower definition of asthma (“epidemiologic definition”) (31), defined as clinician diagnosis of asthma at age 5 years with either asthma medication use or asthma symptoms during age 4–4.9 years (31, 32). (5) We applied the PARS using an alternative cutoff of <6 vs. ≥6. (6) We used asthma outcome variables that more closely approximated those used in each original cohort and calculated the test characteristics of each prediction tool. For API, asthma was defined in this sensitivity analysis as parent report of clinician-diagnosed asthma at age 5 years with at least 1 breathing problem during ages 4–4.9 years, or >3 breathing problems during ages 4–4.9 years regardless of asthma diagnosis (15, 16). For mAPI, asthma was defined in this sensitivity analysis as least one of the following: parent report of clinician-diagnosed asthma at age 5 years; use of inhaled bronchodilator for a breathing problem during ages 4–4.9 years; long-term inhaled corticosteroid use during ages 4–4.9 years; use of inhaled corticosteroids as immediate treatment for a breathing problem during ages 4–4.9 years; and use of oral corticosteroids for a breathing problem during ages 4–4.9 years (17, 18). For PARS, asthma was defined in this sensitivity analysis as parent report of clinician-diagnosed asthma at age 5 years, or at least 1 breathing problem during ages 4–4.9 years regardless of asthma diagnosis (19). Analyses were conducted using Stata 14.0 software (College Station, TX). A two-sided *P* < 0.05 was considered statistically significant.

## Results

In our cohort of 921 infants, parent-reported asthma data were available for 875 (95%). Among these children, 294/875 (34%) had asthma at age 5 years (Table 1). As expected, most characteristics differed by asthma outcome. For example, the prevalence of parental history of asthma was 52% among children who had asthma at age 5 years, compared to 26% among children without asthma (*P* < 0.001).

**Table 1:** Characteristics of children hospitalized for severe bronchiolitis during infancy (MARC-35 cohort), by asthma status at age 5 years.

	**Asthma at age 5 years**			
	**Overall** **(***n*** = 875)**	**No** **(***n*** = 581)**	**Yes** **(***n*** = 294)**	
	***n*** **(%)**	***n*** **(%)**	***n*** **(%)**	***P***
Age at enrollment, months, median (IQR)	3 (2-6)	3 (2-6)	4 (2-6)	<0.001
Male sex	522 (60)	335 (58)	187 (64)	0.09
Race/ethnicity				<0.001
Non-Hispanic white	385 (44)	284 (49)	101 (34)	
Non-Hispanic African-American	199 (23)	106 (18)	93 (32)	
Hispanic	258 (29)	166 (29)	92 (31)	
Other	33 (4)	25 (4)	8 (3)	
African-American race^a^	226 (26)	115 (20)	111 (39)	<0.001
Parental history of asthma reported at enrollment	288 (34)	143 (26)	145 (52)	<0.001
Preterm birth (32–37 gestational weeks)	159 (18)	100 (17)	59 (20)	0.30
Breathing problem history				
Early frequent wheeze (API)^b^	250 (29)	94 (16)	156 (54)	<0.001
Early frequent wheeze (mAPI)^c^	58 (7)	11 (2)	47 (17)	<0.001
Wheezing apart from colds (API, mAPI)^d^	232 (27)	118 (21)	114 (40)	<0.001
Eczema by age 3 years	450 (51)	263 (45)	187 (64)	<0.001
Allergic rhinitis by age 3 years	280 (33)	156 (28)	124 (44)	<0.001
Blood eosinophilia at age 3 years	193 (23)	118 (21)	75 (26)	0.10
Allergic sensitization at age 3 years				
≥1 food allergen	239 (27)	140 (24)	99 (34)	0.003
≥1 aeroallergen	170 (19)	94 (16)	76 (26)	0.001
Polysensitization				0.001
No	417 (48)	290 (50)	127 (43)	
Yes	177 (20)	97 (17)	80 (27)	
Unknown	281 (32)	194 (33)	87 (30)	

*API, Asthma Predictive Index; mAPI, modified Asthma Predictive Index; MARC-35, 35t^h^ Multicenter Airway Research Collaboration; PARS, Pediatric Asthma Risk Score*.

a *Includes African-American children of Hispanic ethnicity*.

b *≥3 breathing problems during age 1–1.9 years or 2–2.9 years*.

c *≥4 breathing problems, all of which lasted >24 h and at least one of which was associated with a healthcare visit, during age 2–2.9 years*.

d *At least one breathing problem without cold during age 1–2.9 years*.

**Figure 1:**
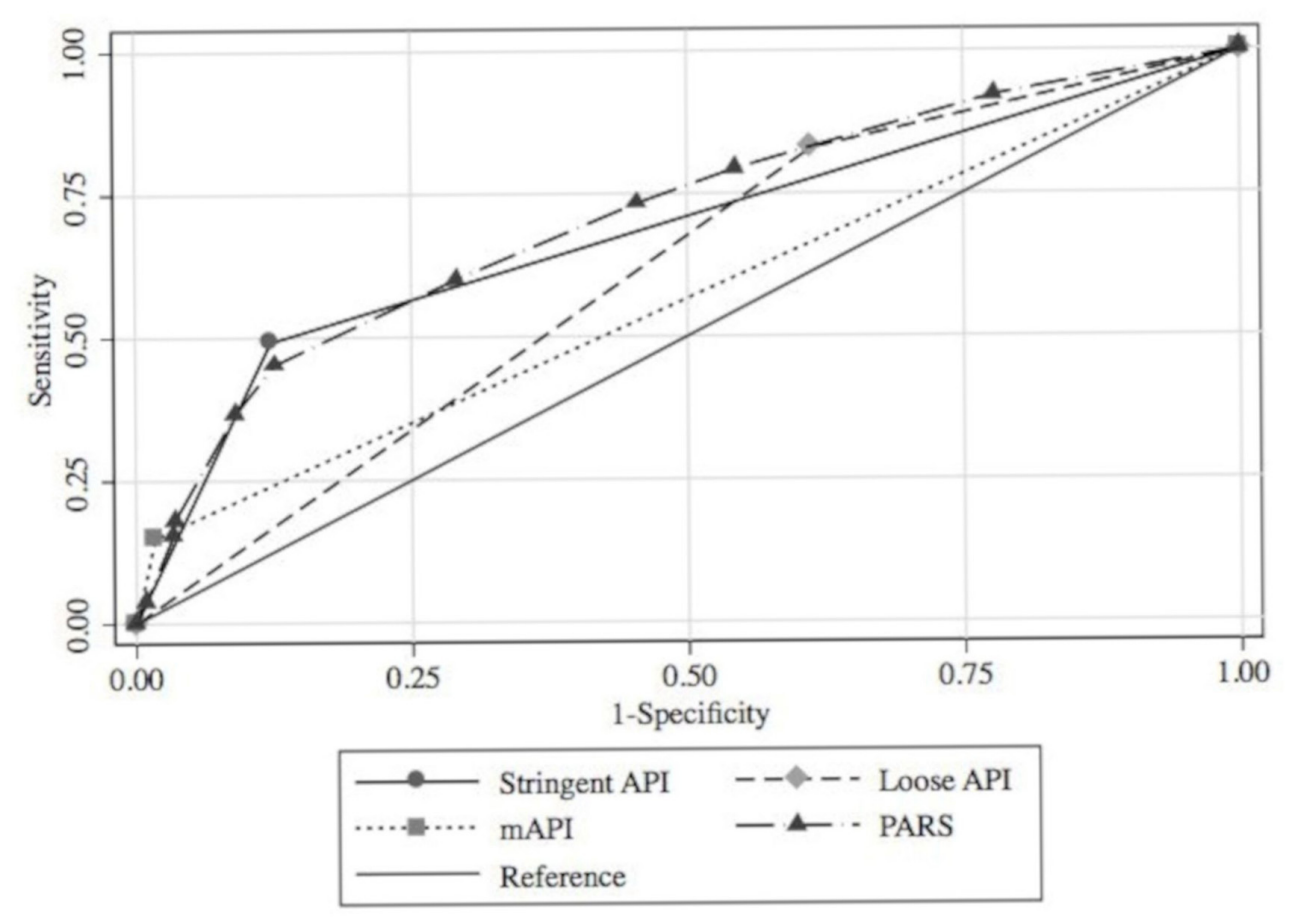
ROC curves for four asthma prediction tools applied to children hospitalized for severe bronchiolitis during infancy (MARC-35 cohort) to predict asthma at age 5 years. MARC-35, 35th Multicenter Airway Research Collaboration; ROC, receiver operating characteristic.

Among the 875 children who had asthma data at age 5 years, sufficient data to calculate the stringent API and mAPI were available for 863 (99%) and 844 (96%), respectively. Sufficient data to calculate the loose API and PARS were available for all 875 (100%). A positive index/score occurred in 24% for stringent API, 68% for loose API, 6% for mAPI, and 55% for PARS (Table 2). The AUCs (95%CI) of the prediction tools applied ranged from 0.57 (95%CI 0.54–0.59) for mAPI to 0.68 (95%CI 0.65–0.71) for the stringent API. The loose API had the highest sensitivity (83%), whereas the mAPI had the highest specificity (98%). The mAPI had the highest LR+ (8.41), while the loose API had the lowest LR– (0.42). The ROC curves of the prediction tools in MARC-35 are shown in Figure 1.

**Table 2:** Performance of API, mAPI, and PARS in predicting asthma development among children hospitalized for severe bronchiolitis during infancy (MARC-35 cohort) and in their original cohorts^a^.

	***n***	**Prevalence of positive index/score,** ***n*** **(%)**	**Sensitivity (%)**	**Specificity (%)**	**PPV (%)**	**NPV (%)**	**LR+**	**LR–**	**AUC (95%CI)**
Stringent API^b^	863	208 (24)	48	88	67	77	3.98	0.59	0.68 (0.65–0.71)
Loose API^b^	875	598 (68)	83	39	41	82	1.37	0.42	0.61 (0.58–0.64)
mAPI^b^	844	52 (6)	15	98	81	70	8.41	0.87	0.57 (0.54–0.59)
PARS^b^	875	479 (55)	73	55	45	80	1.62	0.48	0.64 (0.61–0.67)
	***n***	**Asthma prevalence (%)**	**Sensitivity (%)**	**Specificity (%)**	**PPV (%)**	**NPV (%)**	**LR+**	**LR–**	**AUC**
**Published test characteristics of API, mAPI, and PARS for prediction of asthma development in their original cohorts** ^a^									
Stringent API^c^	937	22	28	96	48	92	7.39	0.75	0.62
Loose API^c^	921	22	57	81	26	94	2.94	0.54	0.69
mAPI^d^	259	28	17	99	90	98	21.0	0.84	N/A
PARS^e^	589	16	68	77	37	93	3.02	0.41	0.80

*API, Asthma Predictive Index; AUC, area under the receiver operating characteristic curve; CI, confidence interval; LR+, positive likelihood ratio; LR–, negative likelihood ratio; mAPI, modified Asthma Predictive Index; MARC-35, 35th Multicenter Airway Research Collaboration; NPV, negative predictive value; PARS, Pediatric Asthma Risk Score; PPV, positive predictive value*.

a *The original cohorts were: API, Tucson Children's Respiratory Study (TCRS); mAPI, Childhood Origins of ASThma (COAST); PARS, Cincinnati Childhood Allergy and Air Pollution Study (CCAAPS)*.

b *The test characteristics of API, mAPI, and PARS in MARC-35 to predict asthma at age 5 years are shown*.

c *The test characteristics of API in TCRS to predict asthma at age 6 years are shown*.

d *The test characteristics of mAPI in COAST to predict asthma at age 6 years using predictor data from age 3 years are shown*.

e *The test characteristics of PARS (cutoff at <7 vs. ≥7) in CCAAPS to predict asthma at age 7 years are shown*.

In their original cohorts, the PARS applied to CCAAPS had the highest sensitivity (68%) and the lowest specificity (77%), while the mAPI applied to COAST had the lowest sensitivity (17%) and the highest specificity (99%) (Table 2). In comparison, the sensitivity and PPV of the stringent API and PARS were higher in MARC-35, while the specificity and NPV were lower. The mAPI had similar sensitivity and specificity in MARC-35 compared to the original cohort, but lower PPV and NPV. The LR+ were consistently lower in MARC-35 than in the published data from the original cohorts.

Across the three prediction tools, the results of the variable importance analysis demonstrated that parental history of asthma had the highest AUC and adjusted OR to predict asthma by age 5 years, followed by wheezing apart from colds. In the API, parental history of asthma was the predictor variable with the highest AUC (0.63) and the highest adjusted OR (OR 2.94, 95%CI 2.10–4.11) to predict asthma at age 5 years (Supplementary Table 2). Eosinophilia had the lowest AUC (0.51) and adjusted OR (OR 1.21, 95%CI 0.78–1.88) among predictor variables in the API. Similar results were observed for the mAPI (Supplementary Table 3). Low AUCs were observed for eosinophilia, aeroallergen sensitization, and food allergen sensitization in mAPI (AUCs 0.53, 0.55, and 0.55, respectively). Among PARS predictor variables, parental history of asthma had the highest AUC and a high adjusted OR (AUC 0.63, OR 2.37, 95%CI 1.61–3.48, Supplementary Table 4). A high adjusted OR was also observed for the African American race (AUC 0.59, OR 2.38, 95%CI 1.58–3.58). Compared to other criteria, polysensitization had a moderate AUC (0.57) and adjusted OR (1.37, 95%CI 0.91–2.07).

The main results were robust to sensitivity analyses (Table 3). Application of the prediction tools to asthma outcomes more similar to those used in the original cohorts generally resulted in similar AUCs, sensitivity, and specificity.

**Table 3:** Test characteristics of API, mAPI, and PARS predicting asthma development at age 5 years among children hospitalized for bronchiolitis during infancy (MARC-35 cohort), in six sensitivity analyses.

	***n***	**Prevalence of positive index/score,** ***n*** **(%)**	**Sensitivity (%)**	**Specificity (%)**	**PPV (%)**	**NPV (%)**	**LR+**	**LR–**	**AUC (95%CI)**
**1. Exclude infants born preterm (32–37 gestational weeks)**									
Stringent API	707	169 (24)	49	88	67	78	4.13	0.58	0.68 (0.65**–**0.72)
Loose API	716	498 (70)	83	37	39	82	1.33	0.45	0.60 (0.57**–**0.64)
mAPI	692	36 (5)	13	99	83	70	10.31	0.88	0.56 (0.54**–**0.58)
PARS	716	390 (54)	72	54	43	80	1.57	0.52	0.63 (0.59**–**0.67)
**2. Complete data for all predictors**									
Stringent API	733	170 (23)	47	89	68	77	4.16	0.60	0.68 (0.64**–**0.71)
Loose API	733	507 (69)	84	38	40	82	1.35	0.43	0.61 (0.58**–**0.64)
mAPI	781	48 (6)	15	98	83	70	10.02	0.86	0.57 (0.55**–**0.59)
PARS	562	330 (59)	77	51	45	81	1.55	0.46	0.64 (0.60**–**0.68)
**3. “Epidemiologic definition” of asthma** ^a^									
Stringent API	847	202 (24)	51	86	59	82	3.78	0.57	0.69 (0.65**–**0.72)
Loose API	859	584 (68)	84	38	34	86	1.35	0.43	0.61 (0.58**–**0.64)
mAPI	830	50 (6)	16	98	74	76	7.56	0.86	0.57 (0.55**–**0.60)
PARS	859	464 (54)	73	53	37	84	1.56	0.50	0.63 (0.60**–**0.67)
**4. Asthma definitions similar to those used in original cohorts**									
Stringent API^b^	847	202 (24)	52	85	53	85	3.48	0.57	0.68 (0.65**–**0.72)
Loose API^b^	859	584 (68)	84	37	30	88	1.35	0.41	0.61 (0.58**–**0.64)
mAPI^c^	844	52 (6)	12	99	90	55	10.14	0.89	0.55 (0.54**–**0.57)
PARS^d^	875	479 (55)	66	60	69	57	1.67	0.56	0.63 (0.60**–**0.66)
**5. Eosinophilia data from early childhood**									
Stringent API	863	212 (25)	48	87	66	77	3.84	0.59	0.68 (0.65**–**0.71)
Loose API	875	606 (69)	84	38	41	83	1.36	0.42	0.61 (0.58**–**0.64)
**6. Cutoff of** **≥6 for PARS**									
PARS	875	547 (63)	79	46	43	81	1.47	0.45	0.63 (0.60–0.66)

*API, Asthma Predictive Index; AUC, area under the receiver operating characteristic curve; LR+, positive likelihood ratio; LR–, negative likelihood ratio; mAPI, modified Asthma Predictive Index; MARC-35, 35th Multicenter Airway Research Collaboration; NPV, negative predictive value; PARS, Pediatric Asthma Risk Score; PPV, positive predictive value*.

a *Parent report of clinician-diagnosed asthma at age 5 years and either asthma symptoms during age 4–4.9 years or asthma medication use during age 4–4.9 years*.

b *Parent report of clinician-diagnosed asthma at age 5 years with at least one breathing problem during ages 4–4.9 years, or >3 breathing problems during ages 4–4.9 years regardless of asthma diagnosis*.

c *At least one of the following: (1) parent report of clinician-diagnosed asthma at age 5 years; (2) use of inhaled bronchodilator for a breathing problem during ages 4–4.9 years; (3) long-term inhaled corticosteroid use during ages 4–4.9 years; (4) short-term use of inhaled corticosteroids as immediate treatment for a breathing problem during ages 4–4.9 years; and (5) use of oral corticosteroids for a breathing problem during ages 4–4.9 years*.

d *Parent report of clinician-diagnosed asthma at age 5 years, or at least 1 breathing problem during ages 4–4.9 years regardless of asthma diagnosis*.

## Discussion

In our analytical cohort of 875 children who had severe bronchiolitis during infancy, 294 (34%) had clinician-diagnosed asthma at age 5 years. This incidence is concordant with previous reports that ~30–40% of infants with severe or recurrent bronchiolitis will develop childhood asthma (11–13, 33). Applied to the MARC-35 cohort, API and PARS had a higher sensitivity and PPV, and lower specificity and NPV, than in their original cohorts. Conversely, mAPI demonstrated similar sensitivity and specificity with lower PPV and NPV. In MARC-35, all tools had a considerably lower LR+. Among the criteria, parental history of asthma and wheezing apart from colds had the strongest associations with asthma at age 5 years.

The API, mAPI, and PARS have notable similarities. These tools combine clinical and laboratory data, including recurrent wheezing episodes, parental asthma, atopic dermatitis, and wheezing apart from colds. These common clinical features are considered risk factors for asthma and are easily measured, justifying their use in prediction tools (12, 14, 17, 19, 21, 34). The main difference between these tools is the objective evidence of type 2 inflammation. While API and mAPI use blood eosinophilia ≥4% (17, 19), mAPI additionally includes sIgE sensitization to milk, egg, or peanuts, and to aeroallergens (19), and PARS uses skin prick test positivity to ≥2 aeroallergens or food allergens (21). Eosinophil levels are correlated with asthma development (35) and recurrent wheezing (36). Serum sIgE sensitization to aeroallergens and food allergens is also correlated to asthma and has been shown to add prediction value (37). Among the three prediction tools, API and PARS use only one objective measure of type 2 inflammation. The stringent API had higher specificity (17, 18) compared to PARS (21, 22) in their original cohorts (18, 22). Additionally, API showed a higher AUC than PARS in MARC-35. PARS had the highest sensitivity in its original cohort (21, 22) and in MARC-35. In contrast, mAPI incorporates two objective measures of type 2 inflammation: eosinophilia and sIgE to foods and aeroallergens (19, 20, 24) mAPI had the highest specificity and LR+ in its original cohort (19, 20, 24) and in MARC-35; however, it had the lowest AUC in MARC-35. Therefore, the prediction contributions of atopic markers alone or in combination are complex and remain undefined. In the analysis of variable importance, objective markers of type 2 inflammation had weaker associations with asthma compared to the other predictors. Markers of type 2 inflammation may provide better discriminatory power in the general population compared to our high-risk cohort of infants hospitalized with bronchiolitis.

Differences in asthma risk and other participant characteristics may contribute to discrepant results between studies. While the API was developed in a healthy infant cohort (18), Caudri et al. (38) demonstrated similar API performance in the high-risk population of children with asthma-like symptoms in the first 4 years of life, in the Prevention and Incidence of Asthma and Mite Allergy birth cohort (39). Rodriguez-Martinez et al. evaluated stringent and loose API performance in a cohort of children with recurrent wheezing (40). Similar to our results, Rodriguez-Martinez et al. found higher sensitivity of the stringent and loose API, but lower specificity and LR+, in their cohort compared to the original cohort. The mAPI was developed in the PEAK trial (19), comprised of children with positive API, and validated in the high-risk population of the COAST study (a birth cohort of infants born to allergic and/or asthmatic parents) (20). PARS was developed in the high-risk CCAAPS (22), a birth cohort of children of atopic parents, and validated in the Isle of Wight unselected birth cohort (21). A higher asthma prevalence was observed in MARC-35 (34%) compared to the TCRS (22%), COAST (28%), and CCAAPS (16%). Accordingly, the PPVs were generally higher in MARC-35 than in their original cohorts, while NPVs were lower. The higher asthma prevalence in MARC-35 may be attributed to the differences in baseline risk among the different cohorts. Thus, despite the apparent applicability of these tools to high-risk populations, there may be unique challenges to the prediction of asthma in children with a history of severe bronchiolitis.

Heterogeneity in asthma outcome definitions may also contribute to the observed differences in asthma prevalence. The TCRS and COAST assessed asthma at age 6 years, whereas CCAAPS evaluated asthma at age 7 years; all used predictor data up to ~age 3 years. We applied the prediction tools using MARC-35 data from infancy to age 3 years to predict asthma diagnosis at age 5 years. Our approach may be expected to improve the performance of the scores, due to greater proximity between the prediction measurements and the outcome; however, this expectation was not borne out. Further discrepancies between asthma definitions exist beyond the timing of assessment. The asthma outcome in TCRS was defined by parent-reported diagnosis and symptoms, while the asthma outcome in COAST was defined by parent-reported physician diagnosis or asthma medication use. The most stringent definition was used in CCAAPS, which required parent report of physician-diagnosed asthma and past-year asthma symptoms, and either >12% increase in FEV_1_ or positive methacholine challenge test result. Thus, the asthma definition used in CCAAPS was more stringent than ours, while the definitions used in TCRS and COAST were arguably less stringent. In a sensitivity analysis, we used asthma definitions that approximated those used in the original cohorts, and observed similar AUCs, sensitivity, and specificity. The PPVs and NPVs were affected, which was expected due to the dependence of these values on the outcome prevalence. Differences in asthma definitions may have partially contributed to the different test characteristics observed in MARC-35 compared to the original cohorts.

Potential limitations include reliance on parent-reported data for multiple factors, including clinician-diagnosed asthma. We recently demonstrated concordance between parent report of clinician-diagnosed asthma and physician documentation of asthma (31). We used parent-reported breathing problems in place of wheezing episodes, as wheezing can only be accurately ascertained through auscultation. This may limit comparability with other studies. For API, the eosinophil values (collected during the bronchiolitis hospitalization) may have been affected by the acute bronchiolitis episode. In API, parents reported physician confirmation of eczema and allergic rhinitis diagnoses, while in MARC-35 parents were not asked specifically if a physician has confirmed these conditions. Therefore, the difference in incidence rates in API and MARC-35 cohorts for eczema and allergic rhinitis may be partially attributable to the different criteria for these conditions (27–29). Allergic sensitization in our cohort was defined by sIgE while skin testing was used in PARS, which may account for some of the observed differences in the determination of allergy sensitization. Asthma was assessed at age 5 years, while TCRS and COAST evaluated asthma at 6 years, and CCAAPS at 7 years which may limit scores' comparability, although we would expect a high predictive accuracy at age 5 years than older given reduction in the period of time between the predictive variables and their outcomes; however, our expectation was not observed. Additionally, it is not known a clear evidence of prediction variables timing dependence leading to asthma development. In API and mAPI asthma prediction accuracy was higher at age 6 years than at age 8 years or older. We did not have airway responsiveness or methacholine test results to use in approximating the CCAAPS asthma outcome. There may be selection bias due to differential loss to follow-up; however, asthma outcome data were available for 95% of the cohort. The study population is composed of infants with severe bronchiolitis (i.e., requiring hospitalization); thus, generalizability beyond this population may be limited.

In conclusion, the three prediction tools performed sub-optimally (AUC <0.8) for predicting asthma at age 5 years in our multicenter, prospective cohort of children with a history of severe bronchiolitis hospitalization during infancy, suggesting the need for a highly accurate (AUC >0.8) asthma prediction tool for children with a history of severe bronchiolitis hospitalization. Further investigations combining clinical, laboratory, and multi-level omics data (e.g., virome, microbiome, metabolome), alongside machine learning techniques, may improve asthma prediction in infants with severe bronchiolitis.

## Data Availability Statement

The original contributions presented in the study are included in the article/Supplementary Material, further inquiries can be directed to the corresponding author. Requests to access the datasets should be directed to Carlos A. Camargo Jr., ccamargo@partners.org.

## Ethics Statement

The studies involving human participants were reviewed and approved by the Institutional Review Board at all participating hospitals. Written informed consent to participate in this study was provided by the participants' legal guardian/next of kin.

## Author Contributions

RC conceptualized and designed the study, collected data, drafted the initial manuscript, and reviewed and revised the manuscript. RG conceptualized and designed the study, coordinated data collection, drafted the initial manuscript, carried out analyses, and reviewed and revised the manuscript. LC collected data, drafted the manuscript, and reviewed and revised the manuscript. JE conceptualized and designed the study, coordinated data collection, supervised data analyses, and reviewed and revised the manuscript. LR and KH interpreted the data and reviewed and revised the manuscript. CC mentored the conceptualization of the study design, data selection, drafted the manuscript, and critically reviewed and revised the manuscript for important intellectual content. All authors contributed to the article and approved the submitted version.

## Funding

This study was supported by the grants U01 AI-087881, R01 AI-114552, and UG3/UH3 OD-023253 from the National Institutes of Health (Bethesda, MD). LR was supported by T32HL116276 from the National Institutes of Health.

## Author Disclaimer

The content of this manuscript is solely the responsibility of the authors and does not necessarily represent the official views of the National Institutes of Health. The National Institutes of Health (NIH) had no rule in the design and conduct of the study.

## Conflict of Interest

The authors declare that the research was conducted in the absence of any commercial or financial relationships that could be construed as a potential conflict of interest.

## Publisher's Note

All claims expressed in this article are solely those of the authors and do not necessarily represent those of their affiliated organizations, or those of the publisher, the editors and the reviewers. Any product that may be evaluated in this article, or claim that may be made by its manufacturer, is not guaranteed or endorsed by the publisher.
