# Supplementary material for: Performance of Three Asthma Predictive Tools in a Cohort of Infants Hospitalized With Severe Bronchiolitis
Source: Front Allergy. 2021 Oct 22;2:758719. doi: 10.3389/falgy.2021.758719 (PMC8974736; doi:10.3389/falgy.2021.758719)
Supplement: Supplementary file 1 [file Table_1.DOCX]

**Table of Contents**

- **Appendix.** Principal Investigators at the 17 participating sites in MARC-35 (page 2)
- **Table E1.** Definitions of the Asthma Predictive Index (API), modified Asthma Predictive Index (mAPI), and Pediatric Asthma Risk Score (PARS) predictor variables, in their original cohorts and in MARC-35 (page 3)
- **Table E2.** Evaluation of API criteria to predict asthma at age 5 years (page 8)
- **Table E3.** Evaluation of mAPI criteria to predict asthma at age 5 years (page 8)
- **Table E4.** Evaluation of PARS criteria to predict asthma at age 5 years (page 9)

**Appendix.** Principal Investigators at the 17 participating sites in MARC-35

| Amy D. Thompson, MD | Alfred I. duPont Hospital for Children, Wilmington, DE |
| --- | --- |
| Federico R. Laham, MD, MS | Arnold Palmer Hospital for Children, Orlando, FL |
| Jonathan M. Mansbach, MD, MPH | Boston Children's Hospital, Boston, MA |
| Vincent J. Wang, MD, MHA and Susan Wu, MD | Children's Hospital of Los Angeles, Los Angeles, CA |
| Michelle B. Dunn, MD and Jonathan M. Spergel, MD, PhD | Children's Hospital of Philadelphia, Philadelphia, PA |
| Juan C. Celedón, MD, DrPH | Children's Hospital of Pittsburgh, Pittsburgh, PA |
| Michael R. Gomez, MD, MS-HCA and Nancy R. Inhofe, MD | Children's Hospital at St. Francis, Tulsa, OK |
| Brian M. Pate, MD and Henry T. Puls, MD | Children's Mercy Hospital & Clinics, Kansas City, MO |
| Stephen J. Teach, MD, MPH | Children's National Medical Center, Washington, DC |
| Stephen C. Porter, MD, MSc, MPH and Richard T. Strait, MD | Cincinnati Children's Hospital and Medical Center, Cincinnati, OH |
| Ilana Y. Waynik, MD | Connecticut Children's Medical Center, Hartford, CT |
| Sujit S. Iyer, MD | Dell Children's Medical Center of Central Texas, Austin, TX |
| Ari R. Cohen, MD, Margaret Samuels-Kalow, MD, MPhil, MSHP, and Wayne G. Shreffler, MD, PhD | Massachusetts General Hospital, Boston, MA |
| Michelle D. Stevenson, MD, MS | Norton Children's Hospital, Louisville, KY |
| Cindy S. Bauer, MD and Anne K. Beasley, MD | Phoenix Children's Hospital, Phoenix, AZ |
| Markus Boos, MD, PhD and Thida Ong, MD | Seattle Children's Hospital, Seattle, WA |
| Charles G. Macias, MD, MPH and Sarah Meskill, MD | Texas Children's Hospital, Houston, TX |

**Table E1.** Definitions of API, mAPI, and PARS predictor variables, in their original cohorts^a^ and in MARC-35

|  | **API** | | **mAPI** | | **PARS** | | |
| --- | --- | --- | --- | --- | --- | --- | --- |
| **Predictor** | **Original cohort (TCRS)** | **MARC-35** | **Original cohort (COAST)** | **MARC-35** | **Original cohort (CCAAPS)** | **MARC-35** |  |
| Early wheeze | Parent report of wheeze at age 2-year or 3-year survey | All participants meet this criterion via their bronchiolitis hospitalization during infancy | N/A | N/A | Parent report of wheeze at age 1-year, 2-year, or 3-year survey | All participants meet this criterion via their bronchiolitis hospitalization during infancy |  |
| Early frequent wheeze | Parent report of ≥3 wheezing episodes in the past year at age 2-year or 3-year survey | Parent report of ≥3 breathing problems during age 1-1.9 years or age 2-2.9 years | Parent report of ≥4 wheezing episodes lasting at least one day in the past 12 months. At least one of these wheezing episodes was confirmed by a physician (per parent report) | Parent report of ≥4 breathing problems lasting at least one day during age 2-2.9 years. The child visited a healthcare provider for at least one of these breathing problems | N/A | N/A |  |
| Wheezing apart from colds | Parent report of wheezing apart from colds at age 2-year or 3-year survey | Parent report of at least one breathing problem which was not related to an acute respiratory infection during age 1-2.9 years | Parent report of wheezing apart from colds | Parent report of at least one breathing problem which was not related to an acute respiratory infection during age 1-2.9 years | Parent report of at least one wheezing episode that did not occur after a cold at age 1-year, 2-year, and 3-year surveys | At least one lifetime breathing problem which was not related to an acute respiratory infection reported by age 3 years |  |
| Eczema | Parent report of physician-diagnosed eczema in the past year at age 2-year or 3-year survey | Parent report of eczema, defined as an itchy scaly rash that comes and goes for at least 6 months, during the first 3 years of life | History of physician-diagnosed eczema | Parent report of eczema, defined as an itchy scaly rash that comes and goes for at least 6 months, during the first 3 years of life | Parent report of 6 or more months of frequent skin scratching and 6 or more months of red, bumpy, or rough dry skin during the first 3 years of life | Parent report of eczema, defined as an itchy scaly rash that comes and goes for at least 6 months, during the first 3 years of life |  |
| Parental history of asthma | Parent report of history of physician-diagnosed asthma in either parent. | Parent report of history of asthma in either parent. | Parent report of history of asthma in either parent. | Parent report of history of asthma in either parent. | Parent report of history of asthma in either parent. | Parent report of history of asthma in either parent. |  |
| Allergic rhinitis | Parent report of hayfever or stuffy, itchy, or runny nose apart from colds, with physician confirmation that the symptoms were due to allergies, in the past year at age 2-year or 3-year survey. | Parent report that the child had ever had hayfever, or sneezing or a runny or blocked nose without a cold, during the first 3 years of life | N/A | N/A | N/A | N/A |  |
| Blood eosinophilia | ≥4% eosinophils in blood specimen collected at mean (SD) age 10.9 (0.6) months | ≥4% eosinophils at index hospitalization (mean [SD] age 4.0 [2.9] months) or in the age 0-0.9 year medical record. If unavailable from the first year of life, we used data from the age 1-2.9 year medical record | ≥4% eosinophils in blood specimen collected at enrollment (age range 24-48 months) | ≥4% eosinophils in blood specimen collected at the early childhood exam (age range 36-59 months). If unavailable from the exam, we used data from the age 1-3.5 year medical record (preferred) or the first year of life | N/A | N/A |  |
| Allergic sensitization |  |  | Aeroallergen sensitization: sensitization to an aeroallergen via skin prick testing at enrollment (age range 24-48 months)  Food allergen sensitization: sensitization to milk, egg, or peanut via skin prick testing at enrollment (age range 24-48 months) | Aeroallergen sensitization: sensitization to an aeroallergen via sIgE or ISAC chip at the early childhood exam (age range 36-59 months). If unavailable from the exam, we used data from the 1-4.9 year medical record (preferred) or infancy  Food allergen sensitization: Sensitization to a food allergen via sIgE or ISAC chip at the early childhood exam (age range 36-59 months). If unavailable from the exam, we used data from the 1-4.9 year medical record (preferred) or infancy | Polysensitization: Sensitization to ≥2 aeroallergens and/or food allergens at ages 1, 2, 3, 4, or 7 years | Polysensitization: Sensitization to ≥2 aeroallergens or food allergens across infancy, early childhood exam, and the age 1-4.9 year medical record. Participants who only had IgE sensitization data from infancy and did not have polysensitization at that time were considered missing for this predictor |  |
| African-American race | N/A | N/A | N/A | N/A | African American race | Parent report of child's race as Black or African American |  |
| **Outcome** | Parent report of physician-diagnosed asthma and ≥1 wheezing episode in the past year, or parent report of ≥3 wheezing episodes in the past year regardless of asthma diagnosis, at ages 6, 8, 11, and 13 years | Parent report of clinician-diagnosed asthma at age 5 years (assessed biennially starting at age 30 months) | Any of the following in the previous year: physician diagnosis of asthma, use of albuterol for coughing or wheezing episodes, use of a daily controller medication, step-up plan, or use of prednisone for asthma exacerbation, at ages 6, 8, and 11 years | Parent report of clinician-diagnosed asthma at age 5 years (assessed biennially starting at age 30 months) | Parent report of past-year asthma symptoms or ever physician diagnosis of asthma, and either >12% increase in FEV1 or positive methacholine challenge test result, at age 7 years | Parent report of clinician-diagnosed asthma at age 5 years (assessed biennially starting at age 30 months) |  |
| **Prediction rule** | Stringent API: Early frequent wheeze and at least 1 of 2 major criteria (parental history of asthma, eczema) or 2 of 3 minor criteria (allergic rhinitis, wheezing apart from colds, eosinophilia)  Loose API: Early wheeze and at least 1 of 2 major criteria or 2 of 3 minor criteria | | Early frequent wheeze and at least 1 of 3 major criteria (parental history of asthma, eczema, aeroallergen sensitization) or 2 of 3 minor criteria (wheezing apart from colds, eosinophilia, food allergen sensitization) | | Parental asthma, eczema, African American race, and polysensitization each contribute 2 points, and early wheeze and wheezing apart from colds each contribute 3 points, for a total score ranging from 0-14, dichotomized at <7 vs. ≥7 | |  |

Abbreviations: API, Asthma Predictive Index; mAPI, modified Asthma Predictive Index; MARC-35, 35^th^ Multicenter Airway Research Collaboration; PARS, Pediatric Asthma Risk Score; SD, standard deviation.

^a^ The original cohorts were: API – Tucson Children’s Respiratory Study (TCRS); mAPI – Childhood Origins of ASThma (COAST); PARS – Cincinnati Childhood Allergy and Air Pollution Study (CCAAPS).

**Table E2.** Evaluation of API criteria to predict asthma at age 5 years

|  | **AUC^a^** | **Adjusted OR (95%CI)^b^** |
| --- | --- | --- |
| Major criteria |  |  |
| Parental history of asthma | 0.63 | 2.94 (2.10-4.11) |
| Eczema | 0.59 | 1.65 (1.18-2.31) |
| Minor criteria |  |  |
| Allergic rhinitis | 0.58 | 1.43 (1.01-2.04) |
| Wheezing apart from colds | 0.60 | 2.61 (1.81-3.76) |
| Eosinophilia | 0.51 | 1.21 (0.78-1.88) |

Abbreviations: API, Asthma Predictive Index; AUC, area under the receiver operating characteristic curve; CI, confidence interval; OR, odds ratio.

^a^ The AUC of each predictor to predict asthma at age 5 years is shown, using all available data.

^b^ Adjusted ORs and 95%CIs are from a model adjusted for all API predictor variables, for the outcome of asthma at age 5 years. Early (frequent) wheeze was not included because this criterion is required for a positive API. A complete case analysis was conducted (model n=733).

**Table E3.** Evaluation of mAPI criteria to predict asthma at age 5 years

|  | **AUC^a^** | **Adjusted OR (95%CI)^b^** |
| --- | --- | --- |
| Major criteria |  |  |
| Parental history of asthma | 0.63 | 2.69 (1.94-3.72) |
| Eczema | 0.59 | 1.66 (1.20-2.30) |
| Aeroallergen sensitization | 0.55 | 1.28 (0.84-1.94) |
| Minor criteria |  |  |
| Wheezing apart from colds | 0.60 | 2.57 (1.81-3.63) |
| Eosinophilia | 0.53 | 1.02 (0.69-1.52) |
| Food allergen sensitization | 0.55 | 1.21 (0.84-1.76) |

Abbreviations: AUC, area under the receiver operating characteristic curve; CI, confidence interval; mAPI, modified Asthma Predictive Index; OR, odds ratio.

^a^ The AUC of each predictor to predict asthma at age 5 years is shown, using all available data.

^b^ Adjusted ORs and 95%CIs are from a model adjusted for all mAPI predictor variables, for the outcome of asthma at age 5 years. Early frequent wheeze was not included because this criterion is required for a positive mAPI. A complete case analysis was conducted (model n=781).

**Table E4.** Evaluation of PARS criteria to predict asthma at age 5 years

|  | **AUC^a^** | **Adjusted OR (95%CI)^b^** |
| --- | --- | --- |
| Parental history of asthma | 0.63 | 2.37 (1.61-3.48) |
| Eczema | 0.59 | 1.40 (0.95-2.05) |
| African American race | 0.59 | 2.38 (1.58-3.58) |
| Polysensitization | 0.57 | 1.37 (0.91-2.07) |
| Early wheeze^c^ | 0.50 | omitted due to collinearity |
| Wheezing apart from colds | 0.60 | 2.05 (1.41-2.98) |

Parental asthma, eczema, African American race, and polysensitization each contribute 2 points to the PARS, and early wheeze and wheezing apart from colds each contribute 3 points.

Abbreviations: AUC, area under the receiver operating characteristic curve; CI, confidence interval; OR, odds ratio; PARS, Pediatric Asthma Risk Score.

^a^ The AUC of each predictor to predict asthma at age 5 years is shown, using all available data.

^b^ Adjusted ORs and 95%CIs are from a model adjusted for all PARS predictor variables, for the outcome of asthma at age 5 years. A complete case analysis was conducted (model n=562).

^c^ All MARC-35 children were considered as having early wheeze due to their bronchiolitis hospitalization in infancy.
